# Nodular Lymphoid Hyperplasia in Children: Recurrent Hematemesis

**DOI:** 10.7759/cureus.34363

**Published:** 2023-01-30

**Authors:** Nurfariza Che Husin, Mohd Shahrulsalam Mohd Shah, Mohd Razin Hassan, Wan Mohd Ruzaimie Wan Mohamad Noor, Mohd Tarmizi Md Nor

**Affiliations:** 1 Department of Surgery, School of Medical Sciences, Universiti Sains Malaysia, Kelantan, MYS; 2 Paediatric Surgery Unit, Department of Surgery, School of Medical Sciences, Universiti Sains Malaysia, Kelantan, MYS; 3 Paediatric Surgery Unit, Department of Surgery, Hospital Raja Perempuan Zainab, Kelantan, MYS

**Keywords:** gastrointestinal endoscopy, duodenum, recurrent hematemesis in children, s recurrent hematemesis in children, lymphoid nodular hyperplasia

## Abstract

Nodular lymphoid hyperplasia (NLH) is a pathology of the gastrointestinal tract that is commonly found in children. Most of its etiology is benign and associated with some underlying causes such as food hypersensitivity, viral or bacterial infection, giardiasis, *Helicobacter pylori* (*H. pylori)* infection, immunodeficiency, celiac disease, and inflammatory bowel disease. It is characterized by the growth of submucosal lymphoid tissue and a mucosal response to different types of noxious stimuli. In this report, we present the case of a child with recurrent hematemesis.

## Introduction

While nodular lymphoid hyperplasia (NLH) is a common disease in children, it is rare in adults. It has predominantly been described in children below 10 years of age [1]. NLH is usually asymptomatic, but sometimes it can cause alarming symptoms in children. Diagnosis is made by upper and lower gastrointestinal endoscopic findings and confirmed with histopathology [1-2]. Although NLH is common in children, it is rarely reported in Malaysia.

## Case presentation

**Figure 1:**
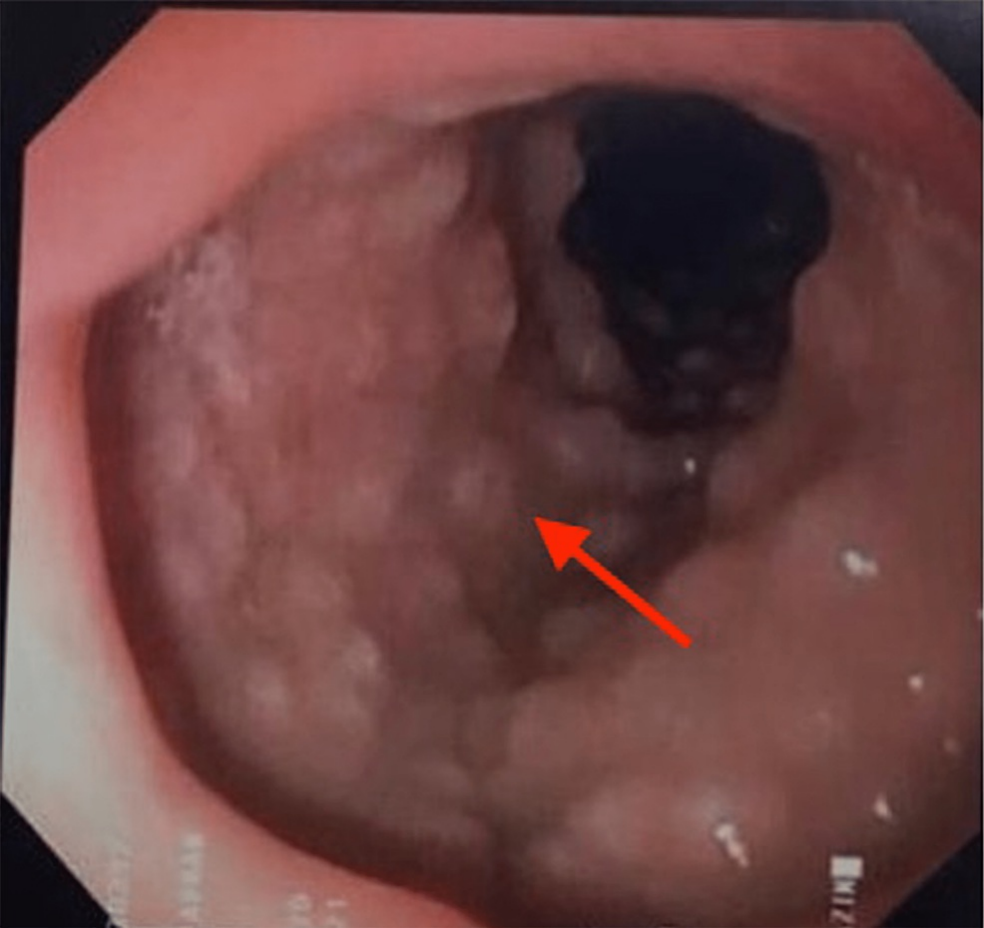
Endoscopic image during EGD showing a circumferential pink-whitish nodular appearance at the duodenum (D1/D2) EGD: esophagogastoduodenoscopy

An eight-month-old female infant presented with milk regurgitation with minimal blood streak for six months. Her symptoms had progressively worsened for the past few weeks before the presentation. She had vomited out one cup of milk curd mixed with mucus and fresh blood once or twice a day after she had taken formula milk. Otherwise, the child had no fever or abdominal pain. She was gaining weight and was active. The clinical examination was unremarkable. Her stool appeared brownish in color. Her blood investigation, stool ova and cyst, and ultrasonography (USG) of the abdomen were normal. Due to recurrent hematemesis, upper esophagogastroduodenoscopy (EGD) was performed. EGD findings showed a diffused circumferential pink-whitish nodular appearance in part 1 until part 2 of the duodenum (Figure 1). A biopsy of the duodenal nodules was taken and sent for histopathology.

**Figure 2:**
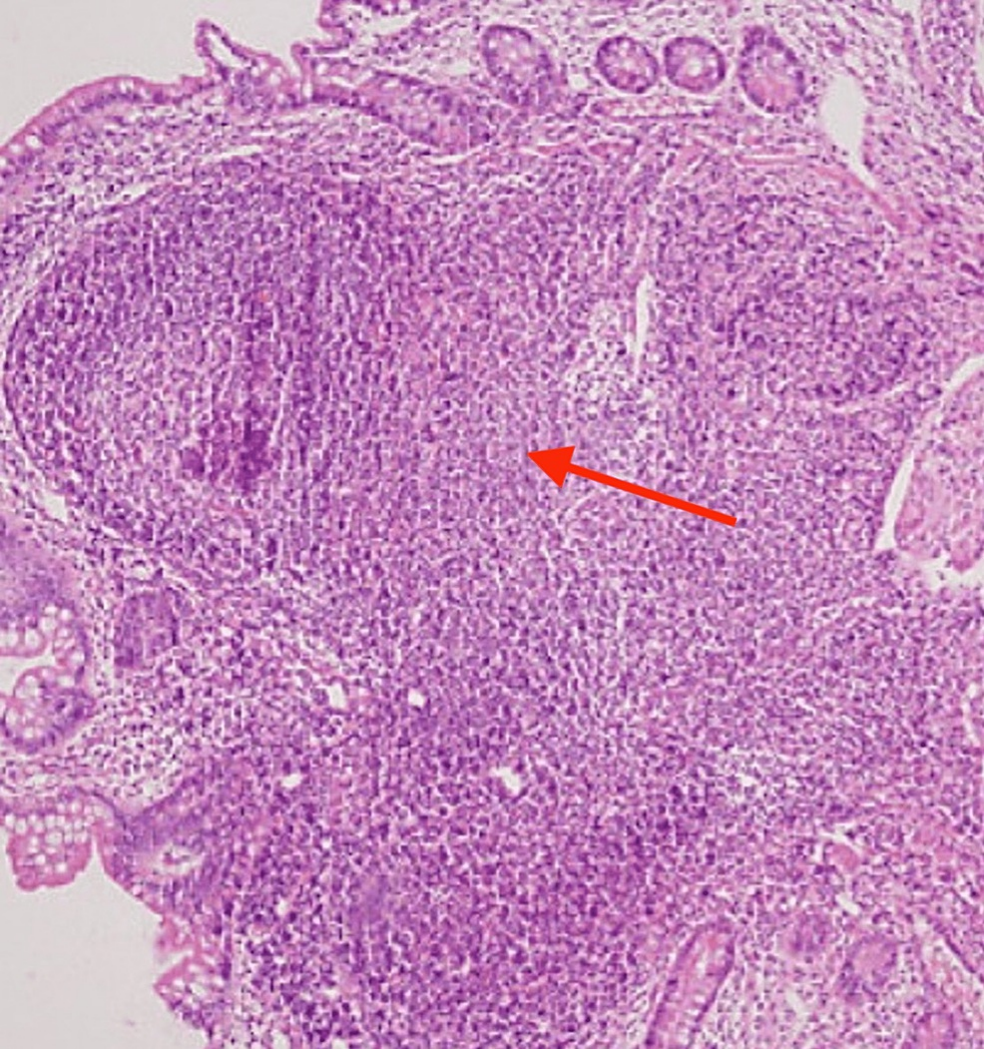
Lymphoid hyperplasia with a well-defined germinal center in the lamina propria

Histopathology findings of duodenal nodules showed a blunting of the villous surface and the presence of lymphoid follicles with well-formed germinal centers located in the lamina propria (Figure 2). Post EGD, the patient remained well and had no more hematemesis. She was treated with an oral proton pump inhibitor (PPI) and anti-reflux medication. We also recommended that the parents change the formula milk and ensure that the feeding bottle is always kept clean.

Upon review during outpatient follow-up, the child looked active, and healthy, and was gaining weight. She did not experience any more hematemesis or vomiting of milk curd after being treated with oral PPI and anti-reflux for six weeks. Hence, the child was given six monthly outpatient follow-ups to review her symptoms and well-being.

## Discussion

Lymphoid follicles are normally found throughout the small and large intestines. In the terminal ileum, these coalesce to form Peyer's patches. It is mainly for the natural self-defense of the gut. NLH is a lymphoproliferative condition caused by a gastrointestinal disease. NLH manifests in two forms: diffuse and focal. In children, about 43% have a lesion in their small bowel and 57% have a lesion in their large bowel. It is usually found in the duodenal bulb, terminal ileum, colon, and rectum. This NLH occurs from the age of three months to 16 years, and the peak incidence is between the ages of six and 10 years [1-2].

The exact pathogenesis of NLH remains unknown, but most of the studies have attributed it to an immunopathological reaction toward the pathogen [3]. NLH has been associated with several diseases, such as food hypersensitivity, viral or bacterial infection, giardiasis, *Helicobacter pylori (H. pylori)* infections, immunodeficiency, celiac disease, and inflammatory bowel disease [2].

The signs and symptoms of NLH vary. Fever and abdominal pain are the most common symptoms (53%), followed by right upper quadrant ‘fullness’ (35%), hematemesis, hematochezia, diarrhea, and weight loss. The complications of NLH in children are intussusception, bowel ischemia, or even fatal colonic torsion [4-6].

NLH can be detected by barium enema study and endoscopy. Classic endoscopic findings include whitish or pinkish diffused nodular lesions 5 to 10 mm in diameter. Endoscopic findings may show gastroesophageal reflux or esophagitis, gastritis, villous atrophy in the small intestine, or colitis [5]. A definitive diagnosis is established by a histopathological finding to differentiate between NLH and other entities like polyposis syndromes such as familial adenomatous polyposis (FAP) and Gardner’s syndrome. The pathognomonic changes associated with NLH are hyperplastic lymphoid follicles with mitotic active germinal centers and well-defined lymphocyte mantles in the mucosa and muscularis propria [3]. 

The treatment of NLH is often directed at an associated condition and its causes. In the pediatric age group, NLH is mainly benign and self-limiting. The eradication of *H. pylori* and giardiasis is usually accompanied by a resolution of symptoms. Regular monitoring of patients with symptoms or endoscopic and biopsy of rapidly growing lesions may prevent the development of lymphomatous transformation.

## Conclusions

NLH is a common condition in children with various clinical manifestations. Its etiology has been linked to the over-regulation of the immune system by response mechanisms of lymphoid tissue associated with the digestive tract. Because of its endoscopic appearance, differential diagnosis requires histopathological confirmation. Thorough history taking, physical examination, and diagnostic endoscopic adjunct will help in establishing a complete diagnosis to aid in its management, thereby reducing the morbidity associated with NLH in children.
